# Perforating Corneal Ulceration in a Patient with Lung Metastatic Adenocarcinoma Treated with Gefitinib: A Case Report

**DOI:** 10.1155/2012/379132

**Published:** 2012-12-18

**Authors:** Enjie Ibrahim, William H. Dean, Nicholas Price, Ahmed Gomaa, Gareth Ayre, Sam Guglani, Ahmed Sallam

**Affiliations:** ^1^Ophthalmology Department, Gloucestershire Hospitals NHS Foundation Trust, Gloucestershire GL53 7AN, UK; ^2^Oncology Department, Gloucestershire Hospitals NHS Foundation Trust, Gloucestershire GL53 7AN, UK

## Abstract

We present a case of a sixty-year-old female who presented with sudden onset of painless loss of vision in one eye due to a perforated corneal ulcer, following three months of treatment with gefitinib, a selective epidermal growth factor receptor (EGFR) tyrosine kinase inhibitor for metastatic adenocarcinoma of the lung with confirmed EGFR gene mutation. The eye did not show any sign of infection or inflammation and had no associated lid problems to account for the development of corneal ulceration. The patient went on to have a corneal graft surgery but postoperatively developed corneal graft melt. This paper aims to raise awareness among ophthalmologists and oncologists of the probable association between gefitinib and corneal ulceration.

## 1. Introduction

Gefitinib (AstraZeneca, UK) is a selective epidermal growth factor receptor (EGFR) tyrosine kinaseinhibitor which has approval in the UK as a first-line treatment of locally advanced or metastatic nonsmall cell lung cancer if they test positive for the EGFR tyrosine kinase mutation [1].

Gefitinib has been previously reported to cause minor ocular surface clinical problems such as tear film dysfunction, meibomianitis, trichomegaly, and trichiasis [2–4]. 

We herein present an unusual case of a patient who presented with a sudden onset of painless loss of vision in one eye following treatment with gefitinib because of perforated corneal ulcer. We aim to highlight the probable association between gefitinib and corneal ulceration. 

## 2. Case Presentation

A 60-year-old Caucasian female patient presented to our eye casualty with a sudden onset of painless loss of vision in the left eye. She had no past history of ocular problems or trauma. Because of metastatic adenocarcinoma lung with the tumour demonstrating a mutation in exon 19 of the EGFR gene, she was considered suitable for primary palliative treatment with gefitinib and has been started on this medication three months earlier. Medical history also included palliative radiotherapy on her spine and right shoulder and treatment with morphine sulphate, diclofenac, and gabapentin. 

**Figure 1:**
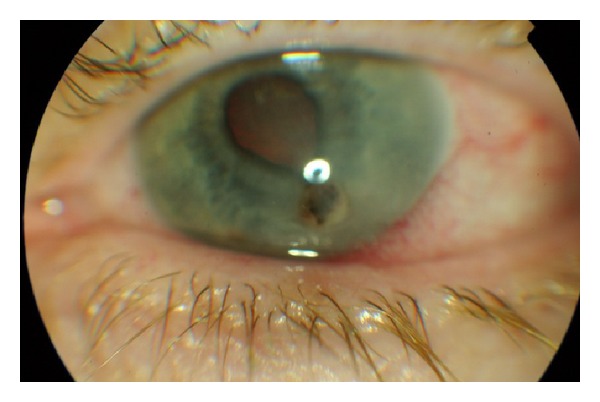
Perforated corneal ulcer plugged by the iris.

On examination, her left eye had very poor vision of perception to light only. Slit-lamp examination revealed a shallow anterior chamber and a perforated corneal ulcer that measured 4.7 × 5 mm, which was plugged by the iris (Figure 1). The eye was white with no conjunctival injection or anterior chamber cells. There was no evidence of meibomianitis or trichomegaly. It was difficult to visualise the fundus due to the corneal pathology but ultrasound examination demonstrated choroidal detachment with no evidence of intraocular metastasis. The right eye showed no abnormality with good vision.

**Figure 2:**
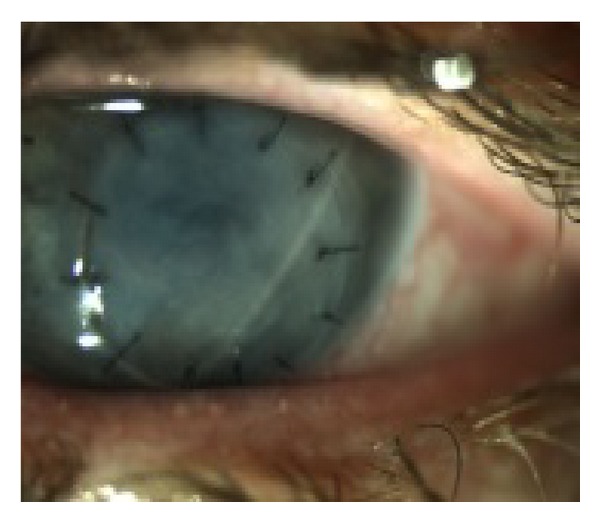
Postoperative appearance following corneal graft surgery with early onset corneal oedema.

As it was suspected that gefitinib could be the cause of her corneal ulcer, her oncologist decided to stop the medication due to the potential ocular toxicity, although gefitinib treatment had reduced her lung tumour size. Initial ophthalmic management included a bandage contact lens to reform the anterior chamber and topical antibiotics. A subsequent tectonic 6.25 mm corneal graft was performed without complication (Figure 2).

**Figure 3:**
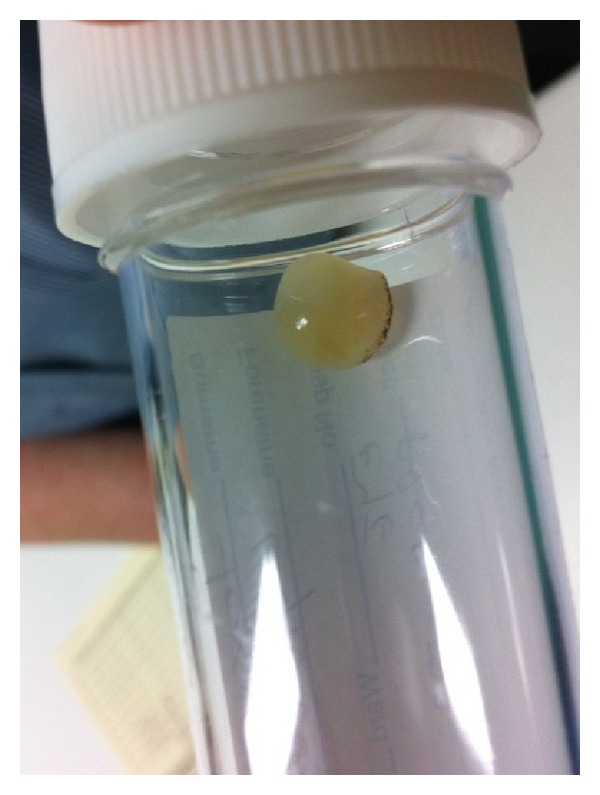
Extruded crystalline lens at the time of melting of the corneal graft.

Post-operatively, her eye remained quiet, however, because of the development of a dense cataract, her vision only improved to hand movement. Two months after stopping the gefitinib treatment and while awaiting cataract surgery, she developed severe left retroorbital pain. On examination, she had iris prolapse through a melting corneal graft, and the crystalline lens was extruded (Figure 3). This was managed initially with botulinum toxin injected into the upper lid producing a ptosis to provide protection for the eye and oral antibiotics were started.

It was agreed, after consultation and counselling, that she was unlikely to benefit from repeat corneal graft surgery mainly because of her general health condition, short life expectancy as well as the potential risk of recurrent graft melt and the eye was left to self eviscerate.

In addition to this, given that her systemic disease was controlled, it was difficult to know whether to continue on the gefitinib, taking into consideration, the potential risks this strategy may pose to her fellow eye. After discussion, her wish was to continue with treatment. She is well with controlled lung cancer and a preserved fellow cornea 10 months later and continues on gefitinib. We have not started the patient on any prophylactic measure to protect the cornea of her healthy eye but we are considering the future use of lubricant eye drops.

## 3. Discussion

This paper describes a patient who developed a perforated corneal ulcer, while on gefitinib treatment; later on, a corneal graft melt possibly due to side effects caused by gefitinib, an EGFR tyrosine kinase inhibitor. 

EGFR is strongly expressed in the basal epithelial cells of the corneal limbus and conjunctiva, and throughout the corneal epithelium, which would most likely explain the side effects associated with gefitinib on the cornea. In preclinical toxicity studies, gefitinib at a dose of 40 mg/kg/day caused reversible thinning of corneal epithelium in both rats and dogs [5]. Furthermore, in dogs treated with the highest dose of gefitinib for 6 months, the corneal opacification was first observed at 1 month progressed on treatment and did not reverse during a 3-month withdrawal period [6]. This may indicate that the effect on the EFGR could persist longer after stopping the medication which may explain the occurrence of corneal graft melt in our case despite drug cessation. Clinically, erlotinib (Roche, UK), another EGFR tyrosine kinase inhibitor, was also found to adversely affect the cornea and has been reported to cause persistent corneal epithelial erosions [7]. 

Although literature research has only came up with dry eyes, [8] and recurrent corneal erosions [7] as possible clinical corneal associations of EGFR inhibitors, the authors believe that gefitinib is a plausible cause of corneal perforation and possibly graft melt in this patient based on the drug effect on the EGFR and the clinical findings of a white eye with no sign of infection or inflammation. It is, however, not clear why only one eye was affected in our patient despite reinitiation of treatment. 

## 4. Conclusion

We recommend that both ophthalmologists and oncologists should be aware of this rare, but potentially sight-threatening, ocular side effect of gefitinib and to report further cases.
